# Autism spectrum disorders: let’s talk about glucose?

**DOI:** 10.1038/s41398-019-0370-4

**Published:** 2019-01-31

**Authors:** Silvia Hoirisch-Clapauch, Antonio E. Nardi

**Affiliations:** 1 Hospital Federal dos Servidores do Estado, Ministry of Health, Av. Atlantica 434 – 1101, Rio de Janeiro – CEP, 22010-000 Brazil; 2 Institute of Psychiatry, Federal University of Rio de Janeiro, Rio de Janeiro, Brazil

## Abstract

Autism spectrum disorders (ASD) are characterized by disconnectivity due to disordered neuronal migration, and by neuronal mitochondrial dysfunction. Different pathways involved in neuronal migration are affected by intrauterine hyperglycemia and hyperinsulinemia, while prolonged neonatal hypoglycemia may cause mitochondrial dysfunction. Our hypothesis was that conditions leading to intrauterine hyperglycemia or neonatal hypoglycemia would influence ASD pathogenesis. In this study, we identified risk factors for ASD by searching PubMed with the MeSH terms “autism spectrum disorder” and “risk factors”. We then analyzed the relationship between the risk factors and glucose abnormalities in the mother and the offspring. The relationship between glucose abnormalities and risk factors such as obesity, excessive maternal weight gain, or diabetes mellitus is evident. For risk factors such as malformations or exposure to selective serotonin reuptake inhibitors, the relationship is speculative. In rodents, for example, intrauterine hyperglycemia is associated with malformations, independent of maternal diabetes. In their turn, selective serotonin reuptake inhibitors reduce the signs of neonatal hypoglycemia. Going undetected, prolonged hypoglycemia may harm the neonatal brain. Importantly, our group demonstrated that either high-carbohydrate diets or physical inactivity the day before delivery may influence neonatal glycemia. In that study, of 158 neonates selected to be screened according to maternal lifestyle risk factors, 48 had hypoglycemia. Of note, five of them had not been identified with current screening programs. Controlled studies are needed to clarify whether maternal interventions aiming at maintaining glycemic control, together with screening programs for neonatal hypoglycemia based on maternal lifestyle risk factors and on exposure to specific prenatal medications can reduce the prevalence of ASD.

## Introduction

Autism spectrum disorders (ASD) are characterized by persistent deficits in social communication and social interaction, as well as by restricted, repetitive patterns of behavior, interests or activities^1^. Such symptoms must be present in the early development period, but may not become fully manifest until social demands exceed limited capacities, or may be masked by learned strategies later in life^1^. Most individuals with ASD have learning disabilities. Structural and diffusion magnetic resonance imaging of ASD brains have consistently shown disrupted neuronal connectivity, due to disordered neuronal migration^2^. Connectivity within the frontal lobe is often excessive and disorganized, while connectivity between the frontal cortex and other brain areas is reduced and unsynchronized^3^.

Neuronal migration starts very early in pregnancy, ending around 26–29 weeks’ gestation, while neuronal connections are formed at five weeks, reaching a peak between weeks 24 and 28^4^. Intrauterine hyperglycemia may affect connectivity through the formation of toxins called advanced glycation end-products^5^, by inhibiting activation of Rac1, a guanosine triphosphatase that regulates neuronal migration^6^ or by modifying the epigenome^7^. Even transient hyperglycemia may cause long-lasting epigenetic changes, which helps explain why rare single nucleotide polymorphisms are prevalent in sporadic ASD^8^ and why concordance for ASD in monozygotic twins is less than 50%^9^.

**Fig. 1:**
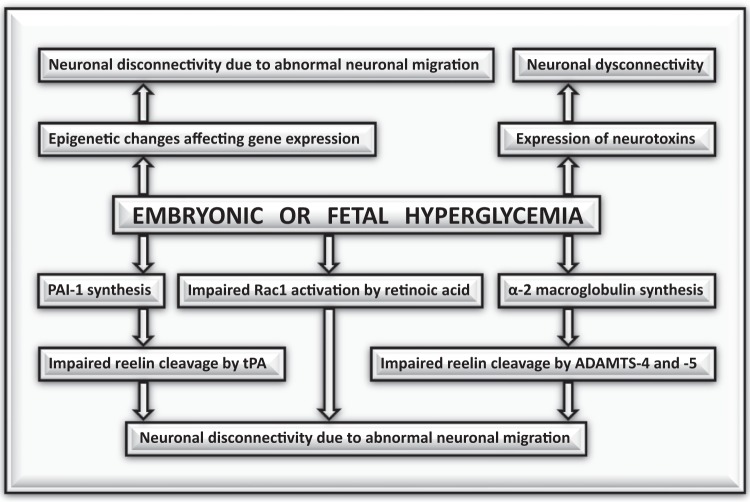
Mechanisms by which intrauterine hyperglycemia may affect neuronal migration and connectivity

Another mechanism by which intrauterine hyperglycemia may affect neuronal connectivity involves reelin, a glycoprotein that guides neurons and glial cells from the ventricular zone to the cortex. Reelin is activated by two proteases known as ADAMTS-4 and -5, and by tissue plasminogen activator (tPA)^10^. Hyperglycemia increases plasma levels of alpha 2-macroglobulin, an inhibitor of ADAMTS-4 and -5^11^, whereas hyperinsulinemia increases plasma levels of plasminogen activator inhibitor (PAI)-1^12^, a major tPA inhibitor. Some authors found no association between ASD and a polymorphism accompanied by elevated PAI-1 levels (PAI-1 4G/5G)^13^, suggesting that the inhibition of ADAMTS-4 and -5, together with tPA inhibition would be required to prevent reelin activation. Figure 1 summarizes the mechanisms by which hyperglycemia may affect neuronal migration and connectivity.

In contrast with the ubiquitous occurrence of disconnectivity, mitochondrial dysfunction—a well-known cause of neurotoxicity—is observed in only 5% of the ASD patients^14^. There are reasons to suspect that the prevalence of mitochondrial dysfunction has been underestimated. This is because neuroimaging performed later in life identifies only chronic mitochondrial dysfunction, such as those related to ATPase mutations, but not transitory mitochondrial dysfunction due to prolonged neonatal hypoglycemia^15^. Of note, our group has shown that current screening programs for neonatal hypoglycemia fail to identify about 10% of the hypoglycemic episodes^16^.

This paper reviews how glucose abnormalities could influence the pathogenesis of ASD. First, it analyzes the relationship between risk factors for ASD and maternal and intrauterine hyperglycemia. Then, it discusses how maternal lifestyle near delivery, by decreasing neonatal glycemia, increases the risk of ASD. Next, it reviews how prenatal medications reported to increase the risk of ASD affect glucose metabolism. Finally, it suggests studies to evaluate whether maternal interventions aimed at maintaining glycemic control, along with new screening strategies for neonatal hypoglycemia, can reduce the prevalence of ASD in populations at risk.

## Risk factors for ASD and intrauterine hyperglycemia

In order to identify risk factors for ASD, we performed a PubMed literature search with MeSH terms “autism spectrum disorders” and “risk factors”. The 680 papers retrieved included risk factors as diverse as maternal obesity, air pollution, vaginal bleeding, preeclampsia, rheumatoid arthritis and the coexistence of malformations (Table 1).

**Table 1:** Risk factors for autism spectrum disorders

	Odds ratio (95% confidence interval)
Maternal diabetes	1.48 (1.25–1.75)^17^^*^
Pre-pregnancy obesity (≥90 kg)	1.69 (1.34–2.14)^21^
Obesity together with gestational diabetes	2.53 (1.72–3.73)^18^
Weight gain of ≥18 kg during pregnancy	1.21 (1.03–1.43)^21^
Weight ≥120 kg at delivery	2.18 (1.51–3.16)^21^
Polycystic ovary syndrome	1.59 (1.34–1.88)^23^
Residence at birth, capital versus rural area	2.35 (2.15–2.57)^25^
Stressful situations (exposure to storms)	3.83 (1.98–7.42)^28^
Preeclampsia	2.36 (1.18–4.68)^32^
Vaginal bleeding	1.81 (1.14–2.86)^36^^*^
Placental insufficiency	5.49 (2.06–14.64)^36^^*^
First-born versus third-born child	1.61 (1.42–1.82)^36^^*^
Pregnancy interval <12 months versus 24–59 months	1.5 (1.28–1.74)^39^
Pregnancy interval >120 months versus 24–59 months	1.44 (1.12–1.85)^39^
Small-for-gestational age babies	2.1 (1.1–3.9)^39^
Mothers with rheumatoid arthritis	1.7 (2.07–2.54)^40^
Mothers with eczema or psoriasis	1.39 (1–1.95)^41^
Asthma treated during pregnancy	1.41 (1.07–1.85)^41^
Children with congenital heart disease	1.97 (1.11–3.5)^42^
Children with cryptorchidism or hypospadias	1.62 (1.44–1.82)^43^
Terbutaline use	1.3 (1.1–1.5)^47^
Selective serotonin reuptake inhibitors use	2.2 (1.2–4.3)^50^
Valproate use	4.4 (2.59–7.46)^57^

The asterisks indicate meta-analyses

The relationship between intrauterine hyperglycemia and risk factors such as diabetes^17–19^ is evident. In a meta-analysis of 12 studies, diabetes mellitus increased by about 50% the risk of having a child with ASD^17^. The risk seems to be higher for type 1 than for type 2 diabetes, and higher for type 2 than for gestational diabetes^19^. When gestational diabetes is diagnosed after 26 weeks of gestation the risk of having a child with ASD is similar to the general population^19^, suggesting that intrauterine hyperglycemia can harm the brain when neurons migrate and connections are formed.

The hypothesis that postprandial hyperglycemia, not diabetes, would be the villain in ASD pathogenesis is reinforced by the finding that prepregnancy obesity combined with gestational diabetes more than doubles the risk of ASD^18^. Aerobic activities are highly effective in normalizing glucose levels, but obese individuals tend to be physically inactive.

Two conditions that may be cause and consequence of postprandial hyperglycemia increase the risk of having a child with ASD: prepregnancy obesity and pregnancy weight gain ≥18 kg^20^. Lack of physical activity combined with a high-carbohydrate diet results in postprandial hyperglycemia and hyperinsulinemia. As insulin is a lipogenic hormone, hyperinsulinemia leads to weight gain and eventually to obesity. In obese individuals, adipose tissue-derived cytokines impair insulin signaling, causing postprandial hyperglycemia^21^.

Additional evidence linking maternal hyperglycemia to ASD comes from a Swedish study with 23,748 ASD cases and 208,796 matched controls, showing that polycystic ovary syndrome increased the risk of ASD by 59%^22^. Given that the prevalence of insulin resistance in polycystic ovary syndrome ranges from 50 to 70%^23^, one could assume that offspring of mothers with polycystic ovary syndrome are often exposed to high-glucose concentrations.

The link between hyperglycemia and ASD risk factors such as urbanicity and pollution^24,25^ seems to involve particulate matter pollutants, which may cause endothelial dysfunction, reducing peripheral glucose uptake^26^. A meta-analysis demonstrated that environmental pollution is associated with alterations in hemoglobin A1c and insulin resistance^26^.

The relationship between intrauterine hyperglycemia and other conditions that increase the risk of ASD is speculative. Some authors have shown a positive relationship between ASD and life stressors such as exposure to hurricanes or tropical storms, or even after marital separation^27,28^. The finding that bereavement does not increase the risk of ASD suggests that the risk of ASD relates to the lifestyle of stressed mothers, not to stress per se^29^. Highly anxious individuals are likely to consume a high-carbohydrate diet^30^, but those experiencing bereavement are usually anorexic.

Pregnancy complications associated with an increased risk of having a child diagnosed with ASD include preeclampsia and placental insufficiency. An American study showed that children with ASD were twice as likely to have been exposed in utero to preeclampsia than controls^31^, while a Swedish group demonstrated that small-for-gestational-age babies have twice the risk of ASD than appropriate-for-gestational-age babies^32^. Maternal hyperinsulinemia has been considered a risk factor for two conditions that may restrict fetal growth: preeclampsia and placental insufficiency^33,34^. Although there is no evidence that maternal lifestyle could increase the risk of vaginal bleeding, which in a meta-analysis doubled the risk of having a child with ASD^35^, pregnant women who bleed are treated empirically with bed rest.

Other conditions that may increase the risk of having a child with ASD include nulliparity^36^ and a short or large interpregnancy interval^36–38^. One could hypothesize that nulliparous expectant mothers and mothers of children younger than 1 year or of children older than 10 years usually spend more time resting than mothers of children aged 2–5 years. The increased prevalence of ASD in children born to mothers with rheumatoid arthritis^39^, psoriasis, eczema, or asthma^40^ may be ascribed to systemic inflammation, to corticosteroid use or to physical inactivity. In a way, all of these could contribute to increase maternal and intrauterine glucose levels.

Different studies have shown that having a malformation increases the risk of being diagnosed with ASD. In Taiwan, the risk of ASD in individuals with congenital heart disease was fivefold that of the general population^41^, while in Israel, the risk of ASD increased by 62% with hypospadias or cryptorchidism^42^. The suspicion that intrauterine hyperglycemia related to high-carbohydrate diets would be a common denominator between malformations and ASD is based on a study showing that intrauterine hyperglycemia is teratogenic, independent of maternal diabetes^43^. In that experiment, the left uterine artery of non-diabetic pregnant rats was infused with saline from 7 to 9 days of gestation, while the left uterine artery was infused with high-glucose concentration. Only embryos exposed to high-glucose concentrations had a high rate of malformations.

## ASD and neonatal hypoglycemia

The mechanisms by which refractory neonatal hypoglycemia (defined as sustained blood glucose <40 mg/dl despite glucose infusion) and severe neonatal hypoglycemia (blood glucose <25 mg/dl) increase the risk of ASD involve energy deprivation and mitochondrial dysfunction^44^. We suspect that undetected hypoglycemia has an important role in ASD pathophysiology for two reasons. One, because many neonates with hypoglycemia are asymptomatic. Two, because it was shown that neonatal hypoglycemia increases threefold the risk of ASD in children born at term, but does not increase the risk in prematures^45^. Preterm neonates are routinely screened for hypoglycemia, but term neonates are not, so it is possible that hypoglycemia in term neonates, going undetected, could harm the brain.

Our group assumed that high-carbohydrate, low-protein diets, and/or physical inactivity would contribute to postprandial hyperglycemia, which would stimulate insulin production in the fetus. Close to delivery, hyperinsulinemia would cause neonatal hypoglycemia^16^. To test the hypothesis that hypoglycemic episodes in the neonatal period may go undetected by current screening programs, we selected neonates born to mothers reporting a high-carbohydrate, low-protein diet (including diabetic mothers requiring >50 g of oral or intravenous glucose to treat iatrogenic hypoglycemia) or physical inactivity within 24 h before delivery. Of the 158 neonates screened for hypoglycemia at 1, 2, and 4 h after birth, 48 had hypoglycemia. All neonates identified by current strategies were identified with the new screening. The reverse was not true: five of the 48 babies diagnosed with hypoglycemia with the new screening were term appropriate-for-gestational-age infants, born to non-diabetic, slim mothers. None of the five was selected to be screened by current strategies^16^.

The hypothesis that undetected neonatal hypoglycemia could increase the risk of ASD is difficult to prove, because once hypoglycemia is identified, strategies to normalize glucose levels are implemented. In the other hand, screening neonates for hypoglycemia based on maternal risk factors—namely physical inactivity and high-carbohydrate intake—could reduce the chances of brain damage related to protracted hypoglycemia.

## Prenatal medications and ASD

It has been reported that intrauterine exposure to terbutaline, to selective serotonin reuptake inhibitors (SSRIs), to valproate, or to high-dose heparin may increase the risk of ASD. Terbutaline, a β2-adrenergic agonist was once used during pregnancy as a bronchodilator or to inhibit uterine contractions. A Danish study, evaluating 5200 ASD children and 55,000 controls, concluded that depending on the trimester, terbutaline exposure increased the risk of ASD by 30–50%^46^. Another study, using an international database, showed that exposure to terbutaline for 2 or more weeks was associated with high ASD concordance in dizygotic twins^47^. Terbutaline crosses the placenta and may cause intrauterine hyperglycemia^48^.

Many authors identified an increased risk of ASD among children exposed intrauterus to SSRIs^49–52^. The risk was independent on depressive symptoms^52^. One relationship between SSRIs and glucose abnormalities is that SSRIs may lead to weight gain^53^, which usually arises from high-carbohydrate intake. Also, these antidepressants are often prescribed with sedatives, and sedated patients tend to exercise less than non-sedated ones. In addition, SSRIs may reduce the signs of neonatal hypoglycemia^54^, preventing its detection. Some authors failed to demonstrate an association between SSRIs and ASD^55^, suggesting that maternal lifestyle related to treatment, not SSRIs themselves, would increase the risk of ASD.

A high risk for ASD has been also reported with valproate, a mood-stabilizer and antiepileptic drug, now contraindicated during pregnancy due to its teratogenicity. A Danish study demonstrated that valproate exposure in utero increased the risk of ASD by 4.4 times^56^. As with SSRIs, valproate is not only associated with carbohydrate craving^57^, but may also reduce the signs of hypoglycemia^58^.

Evidence that high-dose heparin could increase the risk of having a child with ASD comes from a French study, showing that 3 of 36 children born to mothers with antiphospholipid antibody syndrome and none of 12 children born to mothers with systemic lupus erythematosus were diagnosed with ASD^59^. In that study, the prevalence of ASD may have been underestimated, because ASD is usually diagnosed between 2 and 3 years of age, and children were evaluated from 1 to 72 months (mean 11 months). Expectant mothers with antiphospholipid antibody syndrome, but not mothers with lupus without antiphospholipid antibody syndrome, are usually prescribed high-dose heparin, which by improving placental function and preventing prematurity^60^, reduces the chances that neonatal hypoglycemia be detected by current screening programs.

## Future directions

Controlled studies are needed: (i) to compare current screening programs for neonatal hypoglycemia with a screening based on maternal lifestyle risk factors; (ii) to establish the validity of screening for hypoglycemia all neonates born to mothers using SSRIs or high-dose heparin; (iii) to evaluate whether a balanced diet—with or without protein supplementation—along with daily physical activity throughout pregnancy can reduce the prevalence of ASD in populations at risk.

## Conclusions

Glucose abnormalities in the embryo, the fetus or the neonate seem to have a role in the pathogenesis of ASD. Controlled studies are needed to clarify whether interventions aimed at maintaining glycemic control throughout pregnancy, together with new screening programs for neonatal hypoglycemia are effective in reducing the prevalence of ASD.

## Supplementary information

Supplementary Information
